# How women are treated during facility-based childbirth: development and validation of measurement tools in four countries – phase 1 formative research study protocol

**DOI:** 10.1186/s12978-015-0047-2

**Published:** 2015-07-22

**Authors:** Joshua P. Vogel, Meghan A. Bohren, Özge Tunçalp, Olufemi T. Oladapo, Richard M. Adanu, Mamadou Diouldé Baldé, Thae Maung Maung, Bukola Fawole, Kwame Adu-Bonsaffoh, Phyllis Dako-Gyeke, Ernest Tei Maya, Mohamed Campell Camara, Alfa Boubacar Diallo, Safiatou Diallo, Khin Thet Wai, Theingi Myint, Lanre Olutayo, Musibau Titiloye, Frank Alu, Hadiza Idris, Metin A. Gülmezoglu, On behalf of the WHO Research Group on the Treatment of Women During Childbirth

**Affiliations:** Department of Reproductive Health and Research, including UNDP/UNFPA/UNICEF/WHO/World Bank Special Programme of Research, Development and Research Training in Human Reproduction (HRP), World Health Organization, Avenue Appia 20, 1201 Geneva, Switzerland; Department of Population, Family and Reproductive Health, Johns Hopkins Bloomberg School of Public Health, 615 N. Wolfe St, Baltimore, MD USA; School of Public Health, University of Ghana, Accra, Ghana; Cellule de Recherche en Santé de la Reproduction en Guinée (CERREGUI), Enceinte Hôpital National Donka, Conakry, Guinea; Department of Medical Research, Yangon, Myanmar; University of Ibadan, Ibadan, Nigeria; Department of Obstetrics and Gynecology, Korle Bu Teaching Hospital, Korle Bu, Accra, Ghana; Departement de sociologie Université, Sonfonia, Conakry, Guinea; Service de Gynécologie-Obstétrique, Hôpital national universitaire de Conakry, Conakry, Guinea; Maternal and Reproductive Health Division, Department of Public Health, Naypyitaw, Myanmar; Department of Sociology, Faculty of The Social Sciences, University of Ibadan, Ibadan, Nigeria; Department of Health Promotion and Education, Faculty of Public Health, College of Medicine, University of Ibadan, Ibadan, Nigeria; Department of Obstetrics & Gynaecology, Maitama District Hospital, Abuja, Nigeria; Department of Obstetrics & Gynaecology, Nyanya General Hospital, Abuja, Nigeria

**Keywords:** Maternal health, Obstetric delivery, Childbirth, Mistreatment, Disrespect, Abuse, Neglect, Quality of care, Qualitative research

## Abstract

**Background:**

Every woman has the right to dignified, respectful care during childbirth. Recent evidence has demonstrated that globally many women experience mistreatment during labour and childbirth in health facilities, which can pose a significant barrier to women attending facilities for delivery and can contribute to poor birth experiences and adverse outcomes for women and newborns. However there is no clear consensus on how mistreatment of women during childbirth in facilities is defined and measured. We propose using a two-phased, mixed-methods study design in four countries to address these research gaps. This protocol describes the Phase 1 qualitative research activities.

**Methods/Design:**

We will employ qualitative research methodologies among women, healthcare providers and administrators in the facility catchment areas of two health facilities in each country: Ghana, Guinea, Myanmar and Nigeria. In-depth interviews (IDIs) and focus group discussions (FGDs) will be conducted among women of reproductive age (15–49 years) to explore their perceptions and experiences of facility-based childbirth care, focused on how they were treated by healthcare workers and perceived factors affecting how they were treated. IDIs will also be conducted with healthcare providers of different cadres (e.g.: nurses, midwives, medical officers, specialist obstetricians) and facility administrators working in the selected facilities to explore healthcare providers’ perceptions and experiences of facility-based childbirth care and how staff are treated, colleagues and supervisors. Audio recordings will be transcribed and translated to English. Textual data will be analysed using a thematic framework approach and will consist of two levels of analysis: (1) conduct of local analysis workshops with the research assistants in each country; and (2) line-by-line coding to develop a thematic framework and coding scheme.

**Discussion:**

This study serves several roles. It will provide an in-depth understanding of how women are treated during childbirth in four countries and perceived factors associated with this mistreatment. It will also provide data on where and how an intervention could be developed to reduce mistreatment and promote respectful care. The findings from this study will contribute to the development of tools to measure the prevalence of mistreatment of women during facility-based childbirth.

## Background

Worldwide, an estimated 289,000 maternal deaths occurred in 2013, of which 99 % occurred in low- and middle-income countries (LMICs) [1]. While maternal mortality has declined by 45 % since 1990, global progress towards the Millennium Development Goal (MDG) 5 target of a 75 % reduction in the maternal mortality ratio (MMR) has been slow, and many countries will not reach their MDG targets by the end of 2015 [1]. In the past two decades, the rates of skilled birth attendance have steadily increased worldwide [2]. However, 31 % of women still give birth without a skilled attendant present. Increasing skilled birth attendance rates is complex, requiring a comprehensive approach to overcome a range of economic, geographical and infrastructure obstacles to women reaching and utilising facility-based care. Furthermore, greater efforts are needed to ensure that health systems can provide good quality care during childbirth in facilities to all women. Good quality maternal care should be safe, effective, timely and efficient, but also equitable and women-centred [3]. Respect, dignity, equity and emotional support have been identified as essential components of good quality maternal care [3], however these factors are often overlooked or ignored in research and in practice. Greater efforts are needed to define, measure and provide humane, supportive environments in maternity services, in order to ensure that care is delivered in a manner that protects and promotes all women’s rights to dignified and respectful care.

A number of studies on women’s experiences during childbirth suggest that many women encounter poor treatment, neglect, abuse or disrespect from healthcare providers in facilities [4–16]. A recent qualitative systematic review by our group explored facilitators and barriers to facility-based childbirth in low- and middle-income countries. We identified mistreatment, abuse and neglect of women as significant barriers for women to attend facilities for delivery [17]. In 2010, Bowser and Hill published a landscape analysis that explored the evidence for disrespect and abuse during facility-based childbirth and proposed a classification system [18]. As part of the preparatory work for this study, we conducted a mixed-methods systematic review that synthesized qualitative and quantitative evidence of women’s and provider’s perceptions and experiences of mistreatment during childbirth in health facilities globally, to develop an evidence-based typology for the phenomenon [19]. That review identified a range of phenomena that women experienced as mistreatment (see Table 1), including: physical, verbal or sexual abuse, stigma and discrimination, lack of informed consent, breaches of confidentiality, neglect and abandonment, refusal to provide pain relief, a lack of supportive care, detainment in facilities, bribery, and extortion. Women reported being denied food, fluids, freedom to move, as well as preferred (and safe) birthing positions and traditional practices. Health systems factors were also identified as contributing (directly and indirectly) to women’s experiences of mistreatment, such as the poor physical condition of facilities, a lack of necessary equipment, supplies and staff, lack of privacy and a lack of accountability mechanisms. Every woman has the right to dignified, respectful care during childbirth [20–22], and this mistreatment can serve as a powerful disincentive for women to seek care in facilities in the future.

**Table 1:** Typology of the mistreatment of women during childbirth (Reprinted with permission from [19])

Third order	Second order	First order
Physical abuse	Use of force	Women beaten, slapped, kicked, and pinched during delivery
	Physical restraint	Women physically restrained to the bed or gagged during delivery
Sexual abuse	Sexual abuse	Sexual abuse or rape
Verbal abuse	Harsh language	Harsh or rude language
		Judgmental and accusatory comments
	Threats and blaming	Threats of withholding treatment or poor outcomes
		Blaming for poor outcomes
Stigma and discrimination	Discrimination based on sociodemographic characteristics	Discrimination based on ethnicity/race/religion
		Discrimination based on age
		Discrimination based on socioeconomic status
	Discrimination based on medical conditions	Discrimination based on HIV status
Failure to meet professional standards of care	Lack of informed consent and confidentiality	Lack of informed consent process
		Breaches of confidentiality
	Physical examinations and procedures	Painful vaginal exams
		Refusal to provide pain relief
		Performance of unconsented surgical operations
	Neglect and abandonment	Neglect, abandonment and long delays
		Skilled attendant absent at time of delivery
Poor rapport between women and providers	Ineffective communication	Poor communication
		Dismissal of women’s concerns
		Language and interpretation issues
		Poor staff attitudes
	Lack of supportive care	Lack of supportive care from health workers
		Denial or lack of birth companions
	Loss of autonomy	Women treated as passive participants during childbirth
		Denial of food, fluids and mobility
		Lack of respect for women’s preferred birth positions
		Denial of safe traditional practices
		Objectification of women
		Detainment in facilities
Health systems conditions and constraints	Lack of resources	Physical condition of facilities
		Staffing constraints
		Supply constraints
		Lack of privacy
	Lack of policies	Lack of redress
	Facility culture	Bribery and extortion
		Unclear fee structures
		Unreasonable requests of women by health workers

The typology presented in this table is an evidence-based classification system of how women are mistreated during childbirth, based on the findings of the evidence syntheses. The first order themes are identification criteria describing specific events or instances of mistreatment. The second and third order themes further classify these first-order themes into meaningful groups based on common attributes. The third-order themes are ordered from the level of interpersonal relations through the level of the health system

Four recent studies have developed measurement tools based on this classification system, and applied them in facilities in sub-Saharan African countries [23–26]. These studies highlight that many women are being mistreated in facilities during childbirth. However, they have employed different operational definitions and study designs, and report wide differences in prevalence. The tools used in these studies have also not yet been independently validated. It is therefore difficult to determine whether the differences in prevalence relate to differences of methodology or represent true variation.

### Study rationale

With the growing recognition of mistreatment of women during childbirth in facilities, there is a clear need for the development of evidence-based measurement tools that can be applied globally. With these tools, the burden and contributing factors to mistreatment of women at birth can be determined. The tools can also be used to evaluate strategies to prevent and reduce mistreatment of women at birth in facilities. Such efforts are needed to protect women’s fundamental human rights, and as part of strategies to improve quality of care in health facilities.

### How women are treated during facility-based childbirth: development and validation of measurement tools in four countries

We propose using a two-phased, mixed-methods study design in four countries (Ghana, Guinea, Myanmar and Nigeria) to address these gaps. Phase 1 is a formative phase with two specific research activities: a mixed-methods systematic review of the mistreatment of women during childbirth in facilities and a primary qualitative research study. The systematic review has been published [19], in which we proposed a typology for the mistreatment of women across seven domains (see Table 1): (1) physical abuse; (2) sexual abuse; (3) verbal abuse; (4) stigma and discrimination; (5) failure to meet professional standards of care; (6) poor rapport between women and providers; and (7) health systems conditions and constraints.

No single factor can explain why some individuals mistreat or act abusively toward others, or why it is more prevalent in some settings than in others. This has been highlighted in related areas of research (such as research on interpersonal violence) where the ecological framework is often used to understand contributing factors to violence at the individual, relationship, community and societal levels. Similarly, the findings of our review indicate that the mistreatment of women during childbirth is the result of a complex interplay of individual, interpersonal, sociocultural and health system factors. Understanding how these factors relate to how women are mistreated is an important step in the public health approach to preventing its occurrence.

The qualitative research component of Phase 1 will be comprised of in-depth interviews (IDIs) and focus group discussions (FGDs), to explore women’s, healthcare providers’ and health administrators’ experiences and perceptions of how women are treated in health facilities during childbirth. The findings from this formative phase will be used to inform the development of an evidence-based definition, identification criteria and two tools for measuring mistreatment of women during childbirth in facilities: 1) an observation tool, using direct observation of women and providers during labour and delivery in facilities; and 2) a survey tool of women’s postpartum, self-reported experiences of treatment during labour and delivery. In Phase 2, the two tools will be applied in both facility and community settings in the four countries (Ghana, Guinea, Myanmar and Nigeria) for phase 1 but at different study sites. Phase 1 findings will also improve understanding of the individual-, provider- and facility-level factors that may contribute to this mistreatment, and also help identify possible entry points for interventions to promote respectful care and/or reduce mistreatment of women at birth. This study protocol describes the Phase 1 qualitative research activities only. As the findings from Phase 1 will contribute to the conceptualization of Phase 2 operations, the study protocol describing the Phase 2 validation and measurement activities will be published following implementation and analysis of Phase 1 activities.

### Study objectives

The primary objectives of both phases of this research project are:

1. To develop an evidence-based definition and identification criteria of how women are treated during childbirth in facilities;
2. To develop and validate tools for measuring how women are treated during childbirth in facilities;
3. To explore individual, provider, institutional and health systems factors that either promote or prevent respectful or disrespectful practices during childbirth in facilities;

The secondary objectives are:

4. To explore the perspectives and expectations of women, providers and administrators regarding respectful maternal care during childbirth in facilities;
5. To explore the relationship between treatment of women, receipt of biomedical care and individual health outcomes;
6. To understand the relationships between respectful and disrespectful experiences and intended future maternal care-seeking behavior.

Phase 1 activities correspond directly to Objectives 1, 3 and 4, however the findings from Phase 1 will inform Phase 2 activities related to the other objectives. In order to meet these objectives, we have identified a set of six domains that are of specific interest in the Phase 1 qualitative research activities:

- Women’s decision-making processes to choose to deliver at a health facility;
- Women’s expectations of care during childbirth at health facilities, focusing on how they were treated by providers and in the facility environment;
- Women’s, healthcare providers’ and administrators’ experiences and perceptions of treatment during childbirth in health facilities;
- Women’s and healthcare providers’ views of acceptability of mistreatment during childbirth;
- Perceived factors influencing treatment of women during childbirth from the perspectives of women, healthcare providers and administrators; and
- How staff are treated by co-workers and supervisors.

## Methods/Design

### General outline

We will employ qualitative research methodologies among women, healthcare providers and administrators in the facility catchment areas of two health facilities in four countries: Ghana, Guinea, Myanmar and Nigeria (Table 2). We will conduct IDIs and FGDs with women of reproductive age (15–49 years) who reside in the catchment area of selected facilities. We will also conduct IDIs with healthcare providers of different cadres (e.g.: nurses, midwives, medical officers, specialist obstetricians) and facility administrators (e.g.: head of obstetrics and gynaecology department, hospital managers) working in the selected facilities.

**Table 2:** Demographic characteristics of countries participating in phase 1 of the study

	Population [28]	Estimated annual number of births [28]	Skilled birth attendance rate [28]	Maternal mortality ratio [1]
Ghana	25,366,000	794,000	68 %	380
Guinea	11,451,000	428,000	45 %	650
Myanmar	52,797,000	922,000	71 %	200
Nigeria	168,834,000	7,028,000	49 %	560

### Study sites

Four countries were purposively sampled for this study - Ghana, Guinea, Myanmar and Nigeria. These countries were purposively sampled to ensure a range of cultures, languages and settings were captured. In a single region/state within each country, two health facilities were purposively sampled (eight facilities in total). Health facilities in these countries were purposively sampled in collaboration with the country principal investigators with consideration of the following inclusion criteria:

- Secondary level health facility or higher
- 1 rural/peri-urban site, 1 urban site per country
- Well-defined catchment area
- If possible, local facility-based childbirth rate greater than 50 % (in order to minimise selection bias of excluding women who did not deliver in a facility)

### Study participants

Three groups of participants have been identified for this study: (1) women of reproductive age (15–49); (2) healthcare providers working in selected facilities; and (3) facility administrators working in selected facilities. First, to explore individual experiences and perceptions regarding mistreatment during facility-based childbirth, IDIs will be conducted with women of reproductive age who have delivered in any health facility in the past twelve months. Then, to explore community norms regarding mistreatment during facility-based childbirth, FGDs will be conducted with women of reproductive age (15–49 years) who have delivered in any health facility in the past five years. Delivery at a facility in the past five years was selected as inclusion criteria in the FGDs to ensure that women included in this study have ever had an experience of delivering at a facility. Although this excludes women who have never delivered at a health facility (who may have different perceptions of how women are treated during facility-based childbirth), it may include women who have also had recent childbirth experiences outside of a facility. There is a potential for recall bias among this group of participants; however, it is important to capture the perceptions of women who may not have had their last delivery in a health facility in order to reduce selection bias. This sample of women may have important experiences to share, such as how a previous childbirth in a facility influenced their decision to deliver elsewhere.

To explore experiences and perceptions of mistreatment during childbirth, IDIs will be conducted with healthcare providers working in an obstetrical capacity from each of the selected health facilities, including nurses, midwives, medical officers (or other doctors) and obstetricians. To explore facility and health system related factors contributing to mistreatment during childbirth, IDIs will be conducted with facility administrators, such as the head of the hospital or the head of the obstetrics and gynaecology department.

### Participant recruitment

The country principal investigators and country teams will facilitate contact with the women in the communities in the selected facility catchment areas, as well as the healthcare providers and facility administrators in the selected facilities. Potential participants who meet the inclusion criteria will be purposively sampled by the country teams in collaboration with community health workers and hospital outreach coordinators. Each individual will be invited to participate and if they agree, they will be asked to provide informed consent. All FGDs and IDIs will take place in a private setting and will be audio recorded. FGDs and IDIs are anticipated to last between 60 to 90 min and will be conducted by trained moderators from the country teams. Given the sensitivity of the topics to be discussed, all FGD and IDI moderators will be female for the FGDs and IDIs with women participants.

### Sampling

Once the health facilities are selected, a catchment area for each health facility will be defined for study sampling purposes. Purposive sampling will be used to achieve a stratified sample without random selection. This method uses specified parameters (i.e., setting, cadre) to stratify the sample. The sampling grid (Table 3) outlines the stratification proposed for this study. In each country, women will be sampled from urban and rural settings for FGDs and IDIs and will be identified in collaboration with community health workers using community mobilization techniques. Healthcare providers will be sampled based on their cadre, such as nurse/midwives or doctors/specialists. One or two facility administrators per facility will be sampled. We expect the type and title of facility administrator to vary by health facility, but would at minimum include the medical administrative head of the facility and the head of the obstetrics and gynaecology department.

**Table 3:** Sampling strategy to be implemented in each country

	Women of reproductive age (15–49 years) who delivered in any health facility in the past 12 months		Women of reproductive age (15–49 years) who delivered in any health facility in the past 5 years		Facility-based facility-based staff		
*Study site*	*Younger women*	*Older women*	*Younger women*	*Older women*	*Nurses/midwives*	*Doctors/specialists*	*Administrators*
Urban facility and catchment area	8-10 IDIs	8-10 IDIs	2 FGDs	2 FGDs	8 IDIs	8 IDIs	3 IDIs
Rural or peri-urban facility and catchment area	8-10 IDIs	8-10 IDIs	2 FGDs	2 FGDs	8 IDIs	8 IDIs	3 IDIs
Total	32-40 IDIs, women		8 FGDs, women		38 IDIs, providers		

In each country, the sampling strategy will adapted to the local context in consultation with the research teams. This includes, but is not limited to determining the age bracket for “younger” and “older” women and any further stratification for the cadres of participants (e.g.: religion, ethnicity)

### Study instruments

All of the instruments will use the format of semi-structured discussion guides and are available upon request.

IDI guides and FGD guides for women include the following domains:

A. Childbirth narrative
B. Perceptions and experiences of care provided at the most recent facility-based childbirth, focusing on treatment by health workers and the facility environment.
C. Elements and experiences of mistreatment of women during childbirth
D. Perceived factors that influence how women are treated during childbirth
E. Acceptability of how women are treated during childbirth

IDI guides for healthcare providers include the following domains:

A. Childbirth narrative
B. Perceptions and experiences of care provided at the most recent facility-based childbirth, focusing on treatment by health workers and the facility environment.
C. Elements and experiences of mistreatment of women during childbirth
D. Perceived factors that influence how women are treated during childbirth
E. Acceptability of how women are treated during childbirth
F. How staff are treated

IDI guides for facility administrators include the following domains:

A. Perceptions and experiences of care provided at the most recent facility-based childbirth, focusing on treatment by health workers and the facility environment.
B. Perceived factors that influence how women are treated during childbirth
C. How staff are treated

### Project management

This project will be managed by the WHO study coordinating unit, at the WHO Department of Reproductive Health and Research, Geneva, Switzerland. In Ghana, Guinea, Myanmar and Nigeria, the country principal investigators will establish the research teams that will implement the research activities. The study coordinating unit in Geneva will conduct site visits before and during the implementation of the study to contribute to study site selection, training workshops and assessment of adherence to study protocols. Training of country research teams will take place at convenient sites in all countries. There will be continuous communication between country research teams and study coordinating unit at the WHO. Regular contact will be made to ensure that the timeline are followed and problems solved without delay.

### Data management and quality assurance

All qualitative data (FGDs and IDIs) will by digitally recorded and transcribed verbatim in the original language, using a structured transcription format. Transcription will be performed immediately after the IDIs/FGDs are completed to maintain validity of the discussion. Observations and assessments during interviews will be written up as field notes to complement these transcripts. Data transcription will be performed under the supervision of the designated social scientist in each country who will review for completeness. The transcripts in local languages will then be translated into English by an independent translator following the original transcription format. All translated transcripts will undergo another round of consistency checks by the social scientist to maintain high data quality. The social scientists will manage audio and transcribed files and transfer them electronically to the WHO study coordinating unit at regular intervals (weekly or fortnightly). The WHO study coordinating unit will manage all transcript and audio file data. Transcripts will be stored in Atlas.ti computer software on a password-protected computer accessible only to the study team. Transcripts will be de-identified; participants and facilities will be identifiable only by a unique identifier code. Participant’s names and personal information will not be recorded. Transcription and translation will occur in parallel to data collection, and will be shared on an on-going basis with the study team to ensure data quality.

Prior to data collection, a three day training session will be conducted in each country for all research teams, including country principal investigators, data collectors, research assistants, transcribers and translators. The training session will include objectives of the study, data collection procedures, practice sessions and pilot testing with study tools and highlighting ethical considerations. The lead social scientist from each country will ensure that experienced moderators and interviewers are invited to participate in the study. A Manual of Operations will be developed by the WHO study coordinating unit with inputs from country collaborators to standardize the quality of data collection across all countries.

During the data collection period, the country principal investigators will be in constant communication with the interviewers in the field in order to respond to any issues that arise during data collection. Transcripts will be reviewed throughout the data collection process to ensure data content and quality. A random sample of eight transcripts (two per country) will be back-translated into the local language to ensure translation quality.

### Data analysis plan

Thorough debriefing sessions will be conducted between the lead social scientist and the moderators/interviewers on a mutually agreed upon schedule to review field notes, adjust interview guides, and identify potential questions or scenarios of interest or confusion to clarify through member checking in subsequent interviews. We will employ a two-pronged approach for the formal analysis: (1) conduct local analysis workshops with the research team in each country; and (2) line-by-line coding to develop a thematic framework and coding scheme. In each country, a local analysis workshop will be facilitated by the lead social scientist and researchers from the WHO study coordinating unit to review country data. This workshop will also build local capacity by engaging all team members in interpreting project data, as well as permitting sharing of insights from the data collection process and to develop a better understanding of the local context.

The WHO study coordinating unit, in conjunction with the qualitative research teams, will conduct line-by-line open coding on a sample of the translated transcripts to develop the thematic framework. The thematic framework will also be informed by the study objectives to explore women’s, healthcare providers’ and administrator’s experiences and perceptions of mistreatment during childbirth in health facilities, as well as by the typology of mistreatment of women that emerged from the systematic review [19]. The thematic framework will inform the development of a hierarchical coding scheme, which we will apply systematically to all transcripts using Atlas.ti (version 7.1.7 Scientific Software Development GmbH, Eden Prairie, MN). Text units indexed according to each emergent theme will be further analysed and interpreted by the larger study team.

We will explore common themes that span geographic and cultural differences while identifying important differences across settings that need to be accounted for during tool development for the second phase of this project. The measurement tools developed for Phase 2 will take into account themes that are common across study settings, as well as key differences across settings. However, the primary analysis of this study will take into account local issues and context, and will be informed by themes that are most relevant and specific to study settings. Close collaboration between the WHO qualitative research team, the lead social scientists, and the research assistants will ensure quality analysis and interpretation of the data within and across sites.

### Ethical considerations

#### Study population, recruitment strategy and informed consent process

This study will employ broad participation criteria to be as inclusive as possible of all cadres of healthcare providers and women with different life situations (including religious orientation, socioeconomic status, ethnicity, age). Therefore specific sub-groups of healthcare providers or women are not disadvantaged through being unable to participate in the study. Potential participants in the hospital and the community will be identified in collaboration with healthcare providers familiar with the facility and the community. All potential participants will receive information about the study in their language of choice, which will be easy to understand and free of technical jargon. Participants will be given sufficient time to reflect on the information and ask questions. Those who consent to participate in the study will be requested to sign an informed consent form, and it will be made clear that they are free to withdraw from the study at any stage without risk of any negative consequences. For women who cannot read or write, an impartial witness will be present during the entire informed consent reading and discussion. Both the witness and the individual discussing the consent will sign and date the consent form. The contact details of the local investigators, including telephone numbers, will be made available to the participants should they require further information and assistance. Other safeguards will include the use of unique participant numbers on all study forms, and ensuring that interviewers and data collectors are not current or previous employees of the study facility.

#### Perceived risks and benefits of the study

It is possible that women who participate in the semi-structured interviews may become upset if they have experienced mistreatment during childbirth or a traumatic birth experience and the interview revives their feeling of distress. Interviewers will be trained on how to support any woman who becomes upset during the interview, including how to initiate and follow up referral to appropriate section of the hospital where the woman could receive psychological support.

Participants will not experience any direct and/or immediate benefits for participating in the study. However, the study will be gathering information to better inform the development of tools that have the potential to improve the quality of care during childbirth in the future. Study participants and other women using or intending to use facilities for childbirth will benefit from the increased scientific knowledge on this topic, which will ultimately promote women-centred care of high quality in the facilities.

#### Safeguards to protect any recognized vulnerability of the study participants

Vulnerable or potentially vulnerable sub-populations (such as adolescents, women of different ethnicities, migrant women and women who are HIV positive) may participate in this study. We consider it important to ensure that the selection of participants does not discriminate against any group, as women in this category may be at greater risk of receiving poor quality care in the facility. If such women are included, they will be protected by the universal standards of confidentiality and privacy that apply to all participants. However, all women, including these vulnerable groups, will be free to refuse to participate, both confidentially and without prejudice.

#### Reimbursement or compensation to study participants

Women participating in the study will receive a small reimbursement to cover their transportation to the venue of the interview. The value of this payment will be determined in consultation with the country principal investigators, to ensure that it does not constitute an inducement.

#### Responsiveness of the project to community needs and priorities

There is sufficient evidence to suggest that mistreatment of women during childbirth occurs in health facilities worldwide. The findings of this study will be directly applicable to the health facilities and women participating in the research activities. The findings will also inform the development of tools to measure mistreatment of women during childbirth by health providers and to improve the standard of care experienced by women delivering in the health facilities.

#### Deception

There will be no form of deception in this study.

### Gender considerations

All women have a right to respectful care during childbirth and healthcare services need to be structured and organized in a fashion that helps protect and promote those rights. This research aims to improve the identification and measurement of mistreatment of women during facility-based childbirth, so that interventions to prevent and reduce it can be developed and applied appropriately.

This project will reduce gender inequities in two ways. Firstly, this is a public health issue that afflicts women directly, indeed women may be more vulnerable to mistreatment during childbirth compared to other times and contexts. Any efforts to measure and reduce its occurrence will therefore promote gender equity. Secondly, by defining and measuring this mistreatment, it will be possible to identify which sub-groups of women (such as adolescents, women of different ethnicities or other minority groups) are at particular risk. This will further inform and target efforts to ensure that all women receive an equally high standard of respectful care in facilities.

There are some life circumstances for women that will affect their participation in the study. Women who are unable to attend facilities for childbirth (due to economic, geographical or other reasons) will be unable to participate in this study. It is envisaged that the long-term implications of this study's output will be facilitate improvements in the quality of respectful care provided at facilities, and ultimately women's care experiences during childbirth.

This study will involve strong participation of all eligible women, regardless of ethnicity or social status, within the community to achieve the set objectives. Therefore, all community communication and education strategies will be employed to ensure that women within all social strata are invited to participate. These strategies will include the use of posters and leaflets to inform the entire community about the research, interaction with community/opinion leaders, information dissemination at the selected health facilities, and when possible research staff will give talks at community forums and meetings.

#### Ethics approval

The WHO Human Reproduction Programme (HRP) Review Panel on Research Projects (RP2) comprising of external reviewers and WHO scientific staff reviewed and approved the scientific and technical content of Phase 1 of this study (protocol ID, A65880). Ethics approval was obtained from the WHO Research Ethics Review Committee (ERC) and ethics review authorities of all participating sites: Institutional Ethics Review Committee, Department of Medical Research (Lower Myanmar) in Myanmar; Federal Capital Territory Health Research Ethics Committee and Ondo State Ministry of Health Research Ethics Review Committee in Nigeria, Ghana Health Service Ethical Review Committee on Research in Ghana; and the Comite National d’Ethique pour la Recherche en Sante (CNERS) in Guinea.

### Study timeline

The timeframe for the entire project (Phases 1 and 2) is approximately two years. Phase 1 data collection and analysis should be completed over a period of six months. Report writing and results dissemination for Phase 1 and tool development for Phase 2 will occur after Phase 1 data collection and analysis phase is complete.

## Discussion

### Expected study outcomes

The main outcomes of this qualitative study will include an in-depth understanding of: (1) how women are treated during childbirth in health facilities; (2) women’s expectations of care during childbirth; (3) perceived factors that influence the mistreatment of women during childbirth; and (4) how healthcare providers are treated on the maternity wards. These findings will also contribute to the development of two tools to measure the prevalence of mistreatment of women during childbirth. We also anticipate that this formative research will permit identification of potential interventions or strategies to promote respectful care and/or reduce or prevent mistreatment.

### Anticipated problems and proposed solutions

It is possible that participants may not feel comfortable discussing experiences during childbirth in their workplace or other public settings. Interviews will therefore be conducted in a private setting, and the study team will remind participants that their names will not be linked to any responses and encourage the study participants to uphold confidentiality among their peers. Likewise, FGDs will not be conducted among healthcare providers so that colleagues do not disclose personal experiences of witnessing mistreatment.

It is possible that identifying women in the facility-catchment areas, particularly in the urban setting, may be challenging. The study team will rely on the local partners and facility staff in each country to identify the facility-catchment areas from which to identify potential participants. It is also possible that women may not feel comfortable discussing childbirth and/or mistreatment in the FGDs and IDIs. The study team will attempt to mitigate this concern by ensuring the data collectors for FGDs and IDIs are female. It is possible that women who participate in the IDIs may become upset if they have experienced mistreatment during childbirth or a traumatic birth experience and the interview revives their feeling of distress. Interviewers will be trained on how to support any woman who becomes upset during the interview, including how to initiate and follow up referral to appropriate section of the hospital where the woman could receive psychological support.

Any research activity involving violence or mistreatment raises important ethical and safety challenges and safety, confidentiality and interviewer training is very important. We have consulted closely with the WHO ethical and safety recommendations for research on violence against women to guide our research activities on this topic [27]. We have also consulted with research teams from the previous studies [23–25] to determine how these issues were practically managed in other projects. We have addressed each of the eight ethical and safety recommendations for research on violence point-by-point below (Table 4).

**Table 4:** WHO ethical and safety recommendations for research on violence against women

The safety of respondents and the research team is paramount, and should guide all project decisions.	Given the study topic, it is possible that participants and/or the research team can be put at risk of negative or retaliatory responses. In order to reduce this risk, the study, objectives and activities are framed neutrally as a study of women’s experiences of how they are treated during childbirth. This description will be used by research teams to describe the study to others in facilities or in communities, if required.
Prevalence studies need to be methodologically sound and to build upon current research experience about how to minimize the under-reporting of violence.	We have consulted extensively with other researchers on violence against women and mistreatment of women during childbirth to ensure that the study is as methodologically rigorous as possible. In this study women will be asked to disclose information on difficult or painful experiences. Women may feel that these events are too personal, embarrassing or shameful to discuss or feel at risk of reprisal. They may also not recall the event or feel it was not significant. The discussion guides will avoid the use of loaded terms, such as ‘rape’ or ‘violence’ and leading questions regarding how women were treated. The discussion guides will be designed so as to give women multiple opportunities to disclose events of mistreatment or violence during the course of the interview. Adult female interviewers who are not healthcare providers will be sought for the IDIs and FGDs with women in order to enhance women’s disclosure of mistreatment. Interviewers will be carefully selected and appropriately trained in performing the interview, with adequate time and support for piloting and pre-testing the discussion guide. Interviewers will not be permitted to conduct interviews in their own community, or with women known to them.
Protecting confidentiality is essential to ensure both women’s safety and data quality.	In order to ensure confidentiality, the following measures will be taken: (a) all interviewers will receive strict instructions and training on the importance of maintaining confidentiality; (b) participant numbers only will be used to identify women on any study forms; and (c) data will be presented in an aggregated, de-identified manner that does not identify specific women, households or facilities.
All research team members should be carefully selected and receive specialized training and on-going support.	Specialized training will be prepared and provided to all individuals in research teams, focusing on key concepts of mistreatment of women during childbirth. This training will include opportunities to discuss biases, fears and stereotypes, as well as (if relevant) their own experiences during birth. Researchers will be free to withdraw from the project without prejudice. During the data collection phases, regular debriefing meetings will be pre-scheduled to allow the research team to discuss their experiences, feelings and reactions to the study activities. These meetings are intended to reduce stress associated with fieldwork, allow reflection on individual experiences, and identify any possible safety or health concerns that research team members have and avert negative consequences.
The study design must include actions aimed at reducing any possible distress caused to the participants by the research.	Interviewers will be trained to maintain a supportive, non-judgmental manner when conducting interviews, e.g. asking questions in a neutral tone. They will also be trained to anticipate the effect questions may have on the respondent, how to respond to a woman’s level of distress and to recognize the need to terminate an interview if the subject matter becomes too negative. For example, if a woman has had a particularly negative birth experience, it may cause her an emotional distress, necessitating termination of the interview. The discussion will conclude in a positive manner. These standards will be achieved through role-playing activities (observed by trainers) during training.
Fieldworkers should be trained to refer women requesting assistance to available local services and sources of support. Where few resources exist, it may be necessary for the study to create short-term support mechanisms.	Participants in the study may have experienced such mistreatment that they will require additional assistance during or following an interview. In consultation with local research team and local organizations, the research team will identify existing local support services. Once the relevant local services will be identified, they will be contacted and informed of the study, and permission for referral for support obtained.
Researchers and donors have an ethical obligation to help ensure that their findings are properly interpreted and used to advance policy and intervention development.	Findings from this research will be prepared as scientific manuscripts and submitted for publication. Every effort will be made to disseminate and communicate the research findings via formal and informal media locally, nationally and globally. Research findings will also be communicated through WHO and reproductive health research networks. Research findings will be used to inform and strengthen existing advocacy efforts and networks working on women’s rights, reproductive rights and reducing mistreatment of women during childbirth.
Violence questions should only be incorporated into surveys designed for other purposes when ethical and methodological requirements can be met.	The findings from this study will inform the development of tools, for the purposes of measuring mistreatment of women during childbirth.

### Transferability of results

The findings from this study will enhance our understanding of how women are mistreated during childbirth in health facilities, perceived factors that influence this mistreatment and what can be done to improve the treatment provided to women during childbirth. The findings from this study will be further analysed with the thematic framework of the mistreatment of women during childbirth as developed from the systematic review and will contribute to the development of tools to measure the phenomenon. Opportunities for interventions to improve how women are treated during childbirth will also be identified.

### Plans for dissemination of study findings

The results arising from the study will be published in a reputable, open access peer-reviewed journal. All publications will follow relevant external guidance such as the ‘Uniform Requirements for Submission of Manuscript to Biomedical Journals’ issued by the International Committee of Medical Journal Editors (ICMJE). Dissemination of results to participating institutions and communities will take place through meetings of stakeholders within the facilities and the communities and through analysis workshops with local researchers. The results of the study will first be shared with the collaborating investigators. Collaborating investigators will then disseminate local and collective results to their department and relevant authorities within the countries.

## Abbreviations

IDIs: In-depth interviews
FGDs: Focus group discussions
LMICs: Low- and middle-income countries
SBA: Skilled birth attendance
MDGs: Millennium development goals
WHO: World health organization
RP2: Review panel on research projects
HRP: Human reproduction programme
ERC: Ethical review committee
CNERS: Comite National d’Ethique pour la Recherche en Sante
ICMJE: International committee of medical journal editors
